# Improving adrenaline autoinjector adherence: A psychologically informed training for healthcare professionals

**DOI:** 10.1002/iid3.264

**Published:** 2019-07-09

**Authors:** Béré Mahoney, Elaine Walklet, Eleanor Bradley, Steve O'Hickey

**Affiliations:** ^1^ School of Psychology University of Worcester Worcester UK; ^2^ College of Health, Life and Environmental Sciences Worcestershire Acute Hospitals NHS Trust Worcester UK

**Keywords:** adrenaline autoinjector, anaphylaxis, clinician training, patient adherence

## Abstract

**Background:**

Clinicians draw on instructional approaches when training patients with anaphylaxis to use adrenaline autoinjectors, but patient use is poor. Psychological barriers to these behaviours exist but are not considered routinely when training patients to use autoinjectors. Health Psychology principles suggest exploring these factors with patients could improve their autoinjector use.

**Objective:**

To evaluate the impact of a 90‐minute workshop training clinicians in strategies and techniques for exploring and responding to psychological barriers to autoinjector use with patients. Attendees’ knowledge, confidence and likelihood of using the strategies were expected to improve.

**Methods:**

Impact was evaluated using a longitudinal mixed‐method design. Twenty‐nine clinicians (general and specialist nurses, general practitioners, and pharmacists) supporting patients with anaphylaxis in UK hospitals and general practice attended. Self‐rated knowledge, confidence, and likelihood of using the strategies taught were evaluated online 1 week before, 1 to 3, and 6 to 8 weeks after the workshop. Clinicians were invited for telephone interview after attending to explore qualitatively the workshop impact.

**Results:**

*χ* ^2^ analyses were significant in most cases (*P* < .05), with sustained (6‐8 weeks) improvements in knowledge, confidence, and likelihood of using the strategies taught. Thematic analysis of interview data showed the workshop enhanced attendees’ knowledge of the care pathway, understanding of patient's experience of anaphylaxis as psychological not purely physical, and altered their communication with this and other patient groups. However, interviewees perceived lack of time and organisational factors as barriers to using the strategies and techniques taught in clinical contexts.

**Conclusion:**

Training clinicians in psychologically informed strategies produce sustained improvements in their confidence and knowledge around patient autoinjector education, and their likelihood of using strategies in clinical practice.

**Clinical Relevance:**

Exploring psychological barriers should be part of training patients with anaphylaxis in autoinjector use.

## 1 INTRODUCTION

Anaphylaxis is a severe, potentially life‐threatening generalised or systemic hypersensitivity reaction.^1^ ^(p.835)^ International prevalence rates show increasing numbers of children, adolescents, and adults in Europe and the United States are being diagnosed with anaphylaxis.^2^, ^3^, ^4^, ^5^, ^6^, ^7^, ^8^, ^9^, ^10^ However, diagnosis is difficult and anaphylaxis fatalities are probably underreported.^11^ United Kingdom hospital admission data show a population prevalence of 7 per 100 000, a 615% increase from 1992‐2012.^2^ Although a relatively “rare” condition, clinical features of anaphylaxis suggest that prevalence could exceed recorded rates.^11^, ^12^ This, with the known psychological, social, and economic costs to anaphylaxis patients and those supporting them, make effective management of anaphylaxis a research priority.^13^, ^14^, ^15^, ^16^

Long‐term management of anaphylaxis involves educating patients in self‐care behaviours to enable prevention (avoidance of known anaphylaxis triggers), and effective treatment (adherence to a self‐management plan and training to deploy adrenaline with an autoinjector [AAI]).^14^, ^17^ In the event of anaphylaxis, early and appropriate intervention with an AAI is the “first line” treatment against patient death.^3^, ^11^, ^18^, ^19^ Effective use of AAIs requires patient adherence to these behaviours: ensuring the AAI prescription is up to date; carrying an up to date AAI always; accurate recognition of symptoms indicating onset of an anaphylaxis; timely response to these symptoms; and, accurate administration of adrenaline using an AAI. Effective training packages for anaphylaxis patients and their carers to understand and address AAI behaviours are vital for management of this long‐term condition.^20^, ^21^, ^22^

Anaphylaxis patients are typically seen in a range of clinical settings and are routinely in contact with professionals from multiple disciplines including medical doctors, nurses, and pharmacists.^13^, ^18^ Where clinicians provide patient education on AAI use, this has usually been information focused and comprised of practical information and device instructions.^23^, ^24^ Information focused training is necessary but found to be insufficient for effective patient education^25^, ^26^, ^27^ and recent guidelines from the European Academy of Allergy and Clinical Immunology describe current approaches to AAI instruction as ineffective for promoting patient AAI adherence.^13^ Around three‐quarters of clinicians who train patients in AAI use cannot demonstrate the correct technique^18^ and clinicians more broadly do not know how to use AAIs correctly.^5^, ^28^, ^29^ Research suggests barriers to AAI use are not solely practical but incorporate complex psychological features. Patients and clinicians report treatment‐related, psychological, and organisational barriers to AAI adherence.^11^, ^30^, ^31^, ^32^, ^33^ A diagnosis of anaphylaxis can have psychosocial consequences that act as barriers to self‐care behaviours, including effective use of AAIs.^22^ Patients do not routinely update their AAI prescription,^34^ and their carriage and use of AAIs is often poor.^35^, ^36^, ^37^, ^38^, ^39^ Evidence also shows that less than 30% of patients carry their AAI at all times.^4^ Only one‐third to half of patients with anaphylaxis demonstrate the correct use of their AAI, and only 44% report being able to use their AAI correctly.^4^

Specialist allergy staff report feeling ill‐equipped to manage the psychological factors associated with AAI adherence among patients with anaphylaxis, highlighting reasons such as time pressures, limited clinic space, and lack of confidence.^33^ Anaphylaxis self‐management plans have also been found to vary in quality and clinician‐uptake.^40^, ^41^, ^42^ Evidence from paediatric settings has found that staff led AAI training for parents and children can be effective in increasing adherence when it considers complex psychosocial factors which may be barriers to self‐care behaviours, such as the attitudes of family members and carers to anaphylaxis and AAI use, and patients risk perceptions.^36^, ^43^

Behavioural science theories can improve the design of behaviour change interventions.^44^ Theories derived from health psychology could be used to inform AAI training for patients with anaphylaxis to address psychological barriers to AAI adherence. “Training the trainers” of patients, namely clinicians, in psychologically informed strategies for exploring and responding to these barriers with patients could improve clinician delivered training.^45^, ^46^, ^47^, ^48^

This study examined the impact of a psychologically informed workshop designed to improve staff knowledge about psychological barriers to AAI use in patients with anaphylaxis, as well as staff confidence and reported likelihood of using the strategies and techniques taught in their practice.

## 2 METHODS

### 2.1 Design

A mixed‐methods design with a pre‐post questionnaire was used to evaluate the impact of the workshop on participants’ knowledge (of psychological strategies and AAI adherence), confidence (to implemnt strategies), use of strategies and techniques in practice, and organisational barriers to their implementation. Participants completed an online 10‐item forced choice questionnaire before, 1 to 3 and 6 to 8 weeks after attending a workshop (see Table 2 for items).

Qualitative data were collected from attendees in one to one telephone interviews 8 to 10 weeks following the workshop, to provide more in‐depth evaluation of the workshop impact and barriers to implementing the strategies and techniques taught.

### 2.2 Participants

Participant inclusion criteria were:

- They must be a practicing health care professional working in general practice/community health care or hospital‐based service;
- Their clinical role must include prescribing and/or training adults, children or adolescents who have had an anaphylaxis, or their adult carers, to use AAIs.

Clinicians working with patients with anaphylaxis in emergency departments, specialist allergy services, and general/community practice were sampled purposively to ensure workshops groups were multiprofessional. The workshop was delivered on four separate occasions and 29 clinicians attended in total (see Table 1).

**Table 1:** Workshop attendees’ demographic and professional characteristics^a^

Characteristics	
Sex	N = 22 (85%)
Females	N = 4 (15%)
Males	
Age, y (N = 23)	
Range	25‐58
Mean (SD)	40 (11)
Profession	
Medicine	7 (27%)
Nursing	16 (61.5%)
Pharmacy	3 (11.5%)
Years supporting anaphylaxis patients	
Range	0‐30
Mean (SD)	7 (8)
Specialist allergy training	
Yes	7 (27%)
No	19 (73%)
Workshop sites	
General practice	2
Hospital education centre	2

^a^ Data were collected with the online evaluation questionnaire completed before attending the workshop. Although 29 clinicians attended workshops only 26 completed the online evaluation questionnaire before attending that captured these data.

Workshop attendees were mostly female (female: N = 22, 85%; male: N = 4, 15%, *χ* ^2^ = 12.50, *df* = 1, *P* < .001). The majority attending were nurses (N = 16, 61.5%) followed by medical doctors (N = 7, 27%) and pharmacy staff (N = 3, 11.5%), respectively (*χ* ^2^ = 9.00, *df* = 2, *P* = .01). All medical attendees worked in general practice/community health care and all pharmacy clinicians worked in hospital‐based services. Most nurse attendees (N = 10, 62%) worked in general practice/community health care, three (19%) in hospital based paediatric services and three (19%) in hospital emergency departments. Most attendees had no specialist allergy training (no training: N = 19, 73%; specialist allergy training: N = 7, 27%; χ^2^ = 5.60, *df *= 1, *P* = .02).

### 2.3 Materials

#### 2.3.1 Workshop

The workshop was designed to encourage clinicians to use strategies and techniques taught to ask patients about barriers to carrying and using AAIs; and to respond to these using established behaviour change techniques when training patients with anaphylaxis to use AAIs. The workshop was developed using key principles from Intervention Mapping.^49^, ^50^, ^51^, ^52^, ^53^, ^54^, ^55^, ^56^

The 90‐minute training workshop comprised of four parts:

(1) an overview of adherence and why it needs to improve for effective AAI use;
(2) an overview of barriers and facilitators to behaviour change;
(3) theory‐based AAI training using behaviour change techniques to respond to patients’ psychological barriers to AAI adherence;
(4) a group‐based application of the behaviour change strategies and techniques taught using two case studies, followed by reflection on how the strategies taught could be applied within attendees’ clinical practice.

Details of how the workshop was developed can be found elsewhere.^57^ Two documents were produced to support workshop attendees ongoing application of the training in practice: the *Anaphylaxis Management Plan* and the *AAI Training Checklist*. The Checklist describes specific behaviour change techniques (BCTs) clinicians can use when communicating with patients about how they can identify the onset of an anaphylaxis episode, carry and use AAIs effectively and engage in more general self‐management behaviours (eg, AAI prescription renewal and device storage). It is designed to be used as an *aide memoire* for clinicians during health encounters with patients at risk of anaphylaxis (see Appendix for workshop documents).

### 2.4 Procedure

Flyers were distributed at staff meetings to advertise the workshops to eligible clinicians. Interested clinicians were asked to contact the research team for further information. Participants were emailed 1 week before their scheduled workshop, and 1 to 3 and 6 to 8 weeks after attending with a link to the online evaluation questionnaire. Postworkshop emails also invited attendees to contact the research team to arrange a telephone interview in which the workshop impact would be explored further.

### 2.5 Ethics

Ethical approval was granted by the relevant higher education institution, and organisational permission was granted by the relevant National Health Service (NHS) organisation.

## 3 RESULTS

### 3.1 Quantitative evaluation

Ninety percent (N = 26) of the 29 attendees at the four workshops completed the evaluation before attending, dropping to 83% (N = 24; nonresponders: 1 medicine and 1 nursing) at 1 to 3 weeks and 45% (N = 13; nonresponders: 4 medicine and 7 nursing) at 6 to 8 weeks after the workshop. This overall response rate to the online quantitative evaluation is similar to that found in online surveys of clinicians.^58^, ^59^, ^60^

There was no significant difference between the mean age of participants who only completed the online questionnaire before attending the workshop (mean = 40 years, SD = 11) and those who completed the online questionnaire before and on at least one other occasion after attending (mean = 40, SD = 11) (*t*(22) = 0.03, *P* = .90). Likewise, there was no significant difference between the number of males and females in these two groups (*χ* ^2^ with Fisher's exact correction = 1.30, *df* = 1, *P* = .30), although every participant who completed the online questionnaire at all three time points was female. Most participants who completed the online questionnaire before and on at least one other occasion after attending the workshop were nurses (68%, N = 13; medical general practitioners 13%, N = 3; pharmacists 13%, N = 3). Among those who only completed the online questionnaire before attending the workshop, the majority were also nurses (57%, N = 4) and the remainder medical General Practitioners (43%, N = 3).

The quantitative evaluation data were analysed in two ways. *Descriptive statistics* were produced using *χ* ^2^ Tests of Goodness of Fit against expected equal cell sizes (*P* = .05 level). These provide evidence of the overall pattern of clinicians’ self‐reported knowledge, confidence, and intention to use the strategies and techniques taught at each assessment point independent of one another. *Analysis of paired responses* used McNemar's test that compares binary dependent measures at two assessment points and Cochran's *Q* test that compares binary dependent measures at three assessment points. Binary dependent measures were produced by combining the response options for each question with positive or negative meaning (eg, knowledge self‐rated as s*atisfactory*, *fairly good*, *good*, and *very good* were treated as positive, and *very limited* and *don't know* were treated as negative). The McNemar test and Cochran *Q* Test analyses provide evidence of whether clinicians’ self‐reported knowledge, confidence and intention to use the strategies and techniques taught changed positively (improved) across assessment points.

### 3.2 Descriptive statistics

Response frequencies before and at 1 to 3 and 6 to 8 weeks after the workshop were analysed independently using *χ* ^2^ test of Goodness of Fit against expected equal cell sizes (*P* = .05 level). Where cells sizes were small (<5) response categories with similar meaning were combined (eg, *very good* and *good*) to enable inferential statistical analysis. Table 2 shows the number (%) of combined responses, and *χ* ^2^ test results.

**Table 2:** Number (%) of responses to the online evaluation before, 1 to 3 and 6 to 8 weeks after the workshop and *χ* ^2^ analysis results

	Before	1‐3 wk after	6‐8 wk after
How would you rate your knowledge of ways to facilitate AAI adherence with patients?			
Very limited/don't know	15 (58)	2 (8.5)	1 (8)^a^
Satisfactory/fairly good	9 (34)	8 (33.5)	4 (31)
Good/very good	2 (8)	14 (58)	8 (61)
*χ* ^2^(*df*)	9.8 (2)^**^	8 (2)^**^	6.2 (1)^**^
How confident are you in your ability to facilitate AAI adherence in patients?			
Not at all/a little	20 (77)	7 (29)	4 (31)
Moderately/confident/very	6 (23)	17 (71)	9 (69)
*χ* ^2^(*df*)	7.5 (1)^**^	4.2 (1)*	1.90 (1) NS
How frequently do you use strategies to facilitate patients’ adherence to AAI Guidance?			
Never/not very often/don't know	20 (77)	15 (62.5)	9 (69)
Sometimes/most/all the time	6 (23)	9 (37.5)	4 (31)
*χ* ^2^(*df*)	7.5 (1)^**^	1 (1) NS	1 (1) NS
How frequently do you use techniques to improve AAI adherence generally in patients?			
Never/very rarely/rarely	22 (85)	14 (58)	7 (54)
Occasionally	3 (11)	7 (29)	0 (0)^b^
Frequent/very/always	1 (4)	3 (13)	6 (46)
*χ* ^2^(*df*)	31 (2)*	7.8 (2)*	1 (1) NS
Do you intend using strategies to facilitate patient adherence to AAI Guidance in the future?			
Yes	17 (65)	21 (87.5)	11 (85)
No/don't know	9 (35)	3 (11.5)	2 (15)
*χ* ^2^(*df*)	2.5 (1) NS	13.5 (1)^**^	6.2 (1)^**^
Currently, do any issues concern you when dealing with anaphylaxis patients?			
Yes	13 (50)	3 (11.5)	1 (8)
No/don't know	13 (50)	21 (87.5)	12 (92)
*χ* ^2^(*df*)	0 (1) NS	13.5 (1)^**^	9.5 (1)^**^
Do you believe it is possible to bring about changes in patients adherence to AAI guidance?			
None/small number/around half/don't know	16 (61.5)	12 (50)	6 (46)
Most/all	10 (38.5)	12 (50)	7 (54)
*χ* ^2^(*df*)	1.4 (1) NS	0 (1) NS	1 (1) NS
How important is promoting patient adherence to your role as a clinician?			
Not at all/a little/don't know	6 (23)	4 (17)	0 (0)^b^
Moderately/mostly	5 (19)	5 (21)	4 (31)
Very	15 (58)	15 (62)	9 (69)
*χ* ^2^(df)	7 (2)*	9.3 (2)^**^	1.9(2)
Does your work environment enable you to promote patients adherence to AAI Guidance?			
Not at all/a little/don't know	14 (54)	10 (42)	4 (31)
Moderately	10 (38)	8 (33)	0 (0)^b^
Mostly/completely	2 (8)	6 (25)	9 (69)
*χ* ^2^(*df*)	8.7 (2)^**^	1 (2) NS	1.9 (1) NS
Do you have access to resources that could facilitate patients’ adherence to AAI Guidance?			
Yes	6 (23)	18 (75)	10 (77)
No/don't know	20 (77)	6 (25)	3 (23)
*χ* ^2^(*df*)	7.6 (1)^**^	6 (1)^**^	4 (1)*

Abbreviations: *df*, degree of freedom; NS, not significant.

^a^ Due to small cell sizes the *χ* ^2^ test of Goodness of Fit compared very limited/satisfactory (N = 2) and fairly good/good/very good (N = 11) against expected equal cells sizes reducing *df* to 1.

^b^ *χ* ^2^ Test of Goodness of Fit only compared cells containing frequencies against expected equal cell sizes, thus reducing this analysis *df* to 1.

* *P* < .05.

^**^ *P* < .01.

Before the workshop 58% of attendees reported having very limited knowledge about how to facilitate patient AAI adherence. At 1 to 3 and 6 to 8 weeks Postworkshop their self‐reported knowledge had improved, and 58% and 61% respectively now rated their knowledge as *good/very good*. Postworkshop findings also suggest clinician's self‐rated confidence in facilitating patients’ AAI adherence improved from *no/little confidence* (preworkshop) to *moderately/very confident* 1 to 3 and 6 to 8 weeks postworkshop. Participants’ intention to use the strategies and techniques taught to encourage adherence showed sustained improvement in the postworkshop findings, and fewer attendees reported concern about dealing with patients with anaphylaxis (around 10%), a significant change from high rates of concern (50%) preworkshop. However, the number of participants who felt it possible to affect change in anaphylaxis patients’ adherence to AAI guidance showed no significant pre‐post change (pre: 38%; 1‐3 weeks postworkshop 50%; 6‐8 weeks postworkshop 54%). Nevertheless, the percentage of attendees who believed their work environment enabled them to facilitate their patients’ AAI adherence increased following the workshop.

### 3.3 Analysis of paired responses

A series of 2 × 2 McNemar tests (with 0.5 Yates Correction for small cell sizes) were run to compare clinicians’ responses (N = 17) before and 1 to 3 weeks after attending the workshop, and to compare their responses 1 to 3 weeks and 6 to 8 weeks after attending (N = 7). The results showed that at 1 to 3 weeks after compared to before attending the workshop, significantly more clinicians reported that their knowledge about how to facilitate patient AAI adherence (8, *df *= 2, *P* = .05) and their confidence to do so (210, *df *= 2, *P* = .002) had improved. Also, compared to before the workshop, at 1 to 3 weeks after attending more clinicians reported that they were using techniques frequently to encourage patients AAI adherence (3.80, *df* = 2, *P* = .05) and that they had access to resources that could facilitate this (8, *df *= 2, *P* = .005). However, there was no change in clinicians’ self‐reported frequency of using strategies (1.12, *df* = 2, *P* = .30) or their intention to use them (1.12, *df* = 2, *P* = .30). Likewise, compared to before the workshop, at 1 to 3 weeks after attending there were no significant changes clinicians’ concerns about working with patients with anaphylaxis (3, *df *= 2, *P* = .08) or their belief that they could change patients AAI adherence (0.04, *df* = 2, *P* = .90). The importance of promoting adherence to attendees’ clinical role (0.05, *df* = 2, *P* = .80) and how much they believed their work environment enabled them to facilitate patients’ adherence (0.10, *df *= 2, *P* = .80) also remained unchanged across these two assessment points. Comparisons made between clinicians’ responses 1 to 3 weeks and 6 to 8 weeks after attending the workshop were not statistically significant (*P* > .05). This suggests that across the two postworkshop assessment points there were neither significant improvements nor deteriorations in clinicians’ self‐reported knowledge, confidence or intention to use the strategies and techniques taught.

A series of 2 × 3 Cochran *Q* tests were run to compare clinicians’ responses (N = 7) before, 1 to 3 and 6 to 8 weeks after attending the workshop. The results show that those clinicians who completed all three online evaluations reported improved knowledge of ways to facilitate patients’ AAI adherence (*Q* = 6, *df *= 2, *P* = .05) and improved confidence in their ability to do so (*Q* = 8, *df* = 2, *P* = .02). They also reported greater frequency of using strategies and techniques to facilitate patients’ AAI adherence (Q = 6, df = 2, *P* = 0.05; *Q* = 6.5, *df* = 2, *P* = .04), and that they had access to resources that could facilitate patients AAI adherence (*Q* = 7, *df* = 2, *P* = .03).

### 3.4 Qualitative evaluation

All clinical groups attending the workshop were represented in the interviewee sample (see Table 3). Nine interviews were conducted in total (31% of the workshop participant group). Interview duration ranged from 10 to 30 minutes, each digitally audio recorded, transcribed verbatim and anonymised. Thematic analysis (TA) was used to explore interview data.^61^ Themes represent distinct patterns in the data, comprised of smaller information extracts or codes. Codes were identified and collated into themes using a priori interest in the *impact of the workshop on attendees* and their perceptions of *barriers and facilitators to encouraging anaphylaxis patients’ AAI adherence*. Five themes emerged from the codes and these are shown Table 4. The analytic procedure was as follows: working independently, two members of the research team (BM and EW) familiarised themselves with the transcripts, annotating potential codes. Each then analysed the data line‐by‐line to identify codes that enhanced understanding of the a priori issues. Working collectively, BM and EW then collated their codes with similar meaning and then grouped these into themes (see Table 4).

**Table 3:** Interviewee professions

Characteristics	N
Medicine	
Medical general practitioner	1
Nursing	
General practice nurse	1
Allergy service nurse	2
Emergency department nurse	3
Pharmacist	1
Other	
Paediatric play therapist	1

**Table 4:** Interview *a priori* issues, themes and codes

“A Priori” Issue	Theme	Codes
**Impact of the workshop on trainees**	*Transformed clinician knowledge, beliefs and intention*	*Understanding the care pathway*
		*Raised awareness of patient barriers to adherence*
		*Importance of practitioner‐patient communication*
		*Salience of AAI adherence*
		*Knowledge and beliefs about strategies*
		*Intention to change practice*
	*Changed clinician and patient behaviour*	*Improved practitioner‐patient communication strategies*
		*Departmental discussions/disseminating practice*
		*Reinforcement of existing practice*
		*Mirroring staff changes*
		*Patient feedback*
		*Application of behaviour change techniques*
		*Active listening*
		*Practitioner experiences and resources*
**Perception of barriers and facilitators to encouraging anaphylaxis patients AAI adherence**	*Disjointed anaphylaxis care pathway*	*Assumption making*
		*Primary care new ways of working*
		*Adult versus paediatric*
	*Rare condition*	*Clinicians*
		*Patients*
	*Importance of time*	*Time as a barrier*
		*Questioning time as a barrier*
		*Time as a facilitator*
		*Signposting*

Abbreviation: AAI, adrenaline with an autoinjector.

### 3.5 Impact of the workshop on trainees

#### 3.5.1 Transformed clinician knowledge, beliefs, and intention

Interviewees described how they now understood patients’ experiences of anaphylaxis were psychological, not purely physical:

“I didn't appreciate what complications they might have after leaving us… emotions they might go through… it's given me… a broader knowledge of what they might be going through after they've had this event.”

(Emergency Department nurse 1)

Interviewees prior expectations of patients and their adherence were challenged by the workshops and interviewees felt their awareness of adherence behaviour was enhanced. They now understood they should:

“Not just presume because somebody needs something that they're actually going to use it”

(Emergency Department nurse 2)

The workshop heightened interviewees’ awareness of their own role in communicating with patients and the importance of this communication for subsequent patient AAI adherence:

“I didn't realise what the follow‐up and the implications of what we do are.”

(Emergency Department nurse 1)

Several interviewees described how this increased awareness had encouraged them to be more thoughtful about the language they used when sharing information:

“…the odd use of language… might have then led on to a patient's misinterpretation…”

(General Practitioner)

Some reported the workshop had stimulated a fundamental change in their communication style when working with anaphylaxis and other patient groups:

“…a completely different view of how to approach patients now.”

(Pharmacist)

All described becoming sceptical about the effectiveness of instructional information‐driven approaches when training anaphylaxis patients to use AAIs:

“(*the workshop)…* made me think more about not just when you're speaking to people… it isn't just how this works and why you should use it, but thinking about why this person might not use it.…”

(Practice nurse)

The workshop also signalled the important role of all practitioners in encouraging patient AAI adherence, as a mechanism towards better self‐management

“We're clinicians (but) it's really neglectful to give someone that kind of training and not try to influence their behaviour and their thinking, and help them help themselves.”

(Allergy Service nurse 1)

The multiprofessional composition of workshop groups was an unexpected workshop benefit, providing attendees new opportunities for multiprofessional discussion about alternative approaches, referral points and to understand different elements of the care pathway for patients with anaphylaxis. Discussions in workshop groups revealed attendees’ erroneous beliefs about the practices of other healthcare staff working specifically with adult patients with anaphylaxis. Attendees based outside specialist Allergy Services were unaware that, depending on referral circumstances, adult patients particularly may face a lengthy waiting time before being seen within Services. Understanding more about pathway rules and potential wait times encouraged attendees to reconsider the information patients would need about their treatment and prevention of future episodes of anaphylaxis:

“Knowing now, that they're not seen for… months we need to be giving them a bit more time to come to terms with this, and explaining it a little bit more”

(Emergency Department nurse 1)

Allergy service clinicians developed awareness of other clinicians’ assumptions about adult anaphylaxis patients’ care pathway and their own clinical practices with patients:

“People think we do stuff we don't do. We don't see them…”

(Allergy Service nurse 2)

Where participants had attended the workshops as whole teams, aspirations were shared about the potential to change the culture of patient communication and information‐exchange:

“I think, because there were so many of us which were trained at the same time as well, so it now becomes a culture and a general feeling among ourselves.”

(Emergency Department nurse 3)

#### 3.5.2 Changed clinician and patient behaviour

Interviewees described how the workshop increased their confidence and knowledge about exploring and responding to psychological aspects of AAI adherence:

“…in terms of my behaviour, I feel… very confident. Before the workshop I think I scored low there… if I were to encounter these patients… I would be pro‐promoting all of those things.”

(Emergency Department nurse 3)

Many described intending to use the strategies and support documents to enhance different aspects of their clinical practice. Emergency Department clinicians felt the *Anaphylaxis Management Plan* in particular, would help them make better use of the brief health encounters they had with these patients:

“…none of that information was ever given to patients. It might've been verbally said, but… in the heat of A&E and when you're busy, and people have usually had the shock of their lives they're not taking it in… to give them something to go away with, to give them thoughts is excellent…”

(Emergency Department nurse 1)

Changes in clinical practice supporting patients with anaphylaxis had already been made by some clinicians:

“I think I've been more empathetic towards them… when I see them in the hospital and give them their pen, they've had the reaction, and it's either they're first time… all I've done is just bombarded them with questions… now, thinking about it, thinking, “Okay, some… need that support afterwards.”

(Pharmacist)

Interviewees were optimistic that implementing the techniques and strategies learnt would enhance their communication with patients and encourage self‐management behaviours:

“…make(s) them a bit more open to talking to me and telling me things and just being that little bit more open. “Actually, no, maybe I don't take…” If I can be a bit more open… just listening… that was a big thing as well.”

(Emergency Department nurse 2)

Interviewees felt more confident and knowledgeable about how to encourage anaphylaxis patients AAI adherence. Many believed training clinicians in behaviour change and active listening techniques would help them discuss risk and AAI adherence with patients:

“I think with the anaphylaxis thing it's more sort of ‘it's really important that you use this’ …without making people scared.”

(Emergency Department nurse 2)

Discussions with patients using the strategies and techniques taught were described as having the potential to empower patients, encouraging patient's active involvement in shared decision making with staff about their self‐care behaviours:

“It's giving them the tools, things that they might not have thought of, that there might be complications in carrying their EpiPen… It's giving them the tools to think about how they're going to get over those problems.”

(Emergency Department nurse 1)

“I think its… making them aware… their relatives as well… that it's not a disease burden, and it can be prevented. All you need to do is just be confident on using it… knowing where to go… and have that confidence.”

(Pharmacist)

However, there was a perception that this could also depend on clinicians having particular “experience” and “skill.”

“…it's more to do with experience… the more experience I have in talking to patients in that way, will help me develop myself to be a bit more confident on approaching these patients.”

(Pharmacist)

### 3.6 Perceptions of barriers and facilitators to encouraging anaphylaxis patients’ aai adherence

#### 3.6.1 Disjointed anaphylaxis care pathway

Despite interviewees’ optimism about the benefits of using the workshop strategies and techniques, all were aware that anaphylaxis patients could dispense their AAIs from a range of different professionals, and in a range of clinical contexts. Heightened awareness of the importance of communication led to enhanced fears about whether all staff would spend time at administration as required:

“It relies on the nursing staff to counsel the patients as well. Sometimes, when you're just so busy, and you get a script for an *EpiPen*, you just say to the nurse, “Oh, it's stocked on your ward, so just you give it straight from the cupboard”… Hand it over, and then you just hope that they will either read the (instructions)… or the nurse will explain it to them”

(Pharmacist)

All staff were aware of time pressures and the barriers these could place on effective information exchange:

“We see so many patients on… allergy morning clinic… time spent with these patients is a tiny percentage… you're just there to show them the technique, to… get across in five or ten minutes how important it is. Then they're on their way, you never see them again, there's no follow‐up. We don't know if any of them will end up being readmitted.”

(Allergy Service nurse 1)

The workshop facilitated consideration about potential improvements to the anaphylaxis care pathway. Primary care settings were highlighted as providing opportunities for this study:

“…in hospital, they don't really know you. We don't have that relationship because they come in and out. In primary care, they know their doctors… their local community pharmacists, community nurses”

(Pharmacist)

However, some participants felt organisational culture could be a key barrier to any change:

“…it's working with people that don't want change… if the people in charge or the department don't want to make change… there's nothing I can do.”

(Allergy Service nurse 2)

#### 3.6.2 Rare condition

Infrequent encounters were perceived to be a barrier to working consistently with patients to encourage AAI adherence:

“It was a good reminder because we don't actually see very many people with it or deal with it. We often see people with *EpiPens* but people don't have many attacks, do they? We don't have much anaphylaxis.”

(General Practitioner)

The clinical features of anaphylaxis were also felt to be a barrier to AAI adherence:

“…it's the patients themselves as well. If someone had (*not*) an anaphylactic reaction for years, they would just be… “Okay, that's not going to happen to me again…”… they would not be interested. The ones that have had quite a few, would be more interested, or the ones that can remember the reaction, or even relatives that have seen it happen would be more aware and… “Oh, I don't want that to happen again”… patient themselves, if they haven't had it for a while, they won't be that interested… they won't even tell me about their EpiPens on their repeat scripts… they don't think it's important… and “Oh, yes, I just use that when I have a reaction,” that's when I find out more about it… they… don't class it as a medicine… as important for the doctors or the pharmacy team to know.”

(Pharmacist)

For those patients with a first anaphylactic reaction in adulthood, there were perceived to be additional psychological barriers to adherence:

“…it is very different when you're a parent and you're anxious about… your child… parents… say, “I just want to be sure I know how to do this?” they probably want to run through it several times… want a leaflet. Some of them might call back… if you are an adult you've still got that anxiety about giving it to yourself… thinking, “What if I'm already suffering the effects of allergy when I've got to give it.”

(Paediatric Play therapist)

#### 3.6.3 Importance of time

All interviewees perceived time as a primary barrier for them:

“…it's time… the pressure and the stress of working in that environment. If I've got loads of stuff to do, it'd be more difficult to spend a while talking to the patient. Time is precious when you're really stressed… short staffed… getting bleeped everywhere”

(Pharmacist)

Allergy Service staff also described how time could be a barrier in relation to other communication and relationships. Many felt that patients needed more time to feel comfortable describing their emotions and thoughts about their anaphylaxis:

“It's hard to build a professional rapport with someone in that time where they might actually say, “Well, to be honest with you, I'm scared to my wits every time I hear a wasp or a bee.”

(Allergy Service nurse 1)

Some interviewees now believed that clinicians should “make time” given the importance of AAI adherence:

“I feel… you just need to step back and be like, “Okay, I need to make time”.

(Pharmacist)

A number of clinicians believed that invoking time poverty as a barrier reflected organisational culture rather being a real practical barrier:

“It is also, attitude… if you're hardened to not having time, it's’ going to then be very hard to be open to it.”

(Paediatric Play Therapist)

Signposting anaphylaxis patients to other resources that might encourage their AAI adherence was also believed to be a strategy for using clinical time productively:

“Even if you do have ten minutes and… struggling… that's when… to signpost… say ‘I acknowledge that this might be an issue. This is where you can go if you want to find out more.’… may be… there isn't really somewhere else, but even if you're just told “come back again” or “you can call this helpline,” something.”

(Paediatric Play Therapist)

## 4 DISCUSSION

Evidence from paediatric settings shows staff led AAI training that considers complex psychosocial factors associated with self‐care behaviours can be more effective for increasing AAI adherence. The findings of the study reported are consistent with this literature. The workshop improved attendees’ self‐reported knowledge, confidence and likelihood of using the strategies and techniques taught for exploring and responding to patients’ psychological barriers to AAI use. The mixed‐methods longitudinal design suggests improvements were sustained. To the best of our knowledge this is one of a small number of published studies that has used Health Psychology intervention mapping principles to design a brief training intervention for clinicians in psychologically ‐ informed strategies they can use to encourage AAI adherence among adult patients with anaphylaxis.

The quantitative findings are consistent with those from studies that demonstrate training delivered through a lecture plus practical activity workshop format is effective for improving clinician AAI use; and, to educate parents of children with anaphylaxis in effective management of their child's anaphylaxis.^45^, ^46^, ^47^ Clinicians self‐reported knowledge, confidence and intention to use the strategies taught improved after attending the workshop. Evidence shows these are precursors to behaviour change^52^, ^53^, ^54^, ^55^ and clinicians are more likely to engage in education and treatment behaviours with patients if they feel knowledgeable and confident about using the behaviours and report an intention to apply them in their practice.^62^, ^63^, ^64^ Evidence also shows that patients with long‐term conditions have fewer emergency department admissions if supported by clinicians educated in communication strategies of the type taught in the workshop that encourage discussion with clinicians about self‐management concerns and goals.^65^ Patients also prefer clinicians to incorporate the type of strategies and techniques taught in the workshop in their communication with patients (eg, open questions to encourage exploration of self‐care barriers and identifying solutions through shared decision making with clinicians). The strategies and techniques taught in the workshop can also facilitate clinicians use of patient‐led and focused approaches to communication that are preferred by individuals with long‐term conditions, such as anaphylaxis.^48^ Additionally, the strategies and techniques taught in the workshop are designed to encourage patients to take an active role in their AAI education, and patient education provided by clinicians is more effective when the patient takes this role in the process.^66^, ^67^

The workshop impact on how the clinicians interacted with anaphylaxis and other patient groups was an unexpected positive outcome. This shows the workshop was also effective for altering clinicians’ approach to patient education more generally. This new approach incorporated awareness that the provision of information alone is insufficient for facilitating patient's self‐care.^67^

The workshop was less successful in increasing the number of clinicians who believed they could affect change in patients AAI adherence. Pessimism about a behaviour or treatment strategy can be a barrier to its implementation by clinicians.^68^ The qualitative data suggest this pessimism was related to perceived organisational, care‐pathway and time barriers that were perceived to be beyond the control of participants attending the training. The workshop content did not consider these barriers; and, evidence shows clinicians often cite these factors as barriers to implementing new communication strategies with patients in their practice.^68^, ^69^, ^70^, ^71^ The pessimism may, however, also reflect an increased awareness of the complex psychological features associated with anaphylaxis amongst workshop participants. This highlights the importance of providing organisationally relevant signposting and referral pathways for clinical staff who may want to refer patients on for more complex psychological issues associated with anaphylaxis. In planning future workshops, it may be possible to include such information alongside the checklist. In addition, participants could be encouraged to form peer‐to‐peer communities of practice as they implement new strategies in practice, to feedback new ideas as well as unpick ongoing organisational and professional challenges associated with their contact with anaphylaxis patients. These could be supported with virtual platforms or arranged via a 6‐monthly workshop catch‐up group.

The study has several implications for clinical practice. Health Psychology theory can support the development of effective brief training packages to help clinicians to effectively exchange information with patients living with potentially life‐threatening diseases which require patient‐led management and control. A recommendation from this study is that psychologically informed training, with accompanying tools (eg, checklists) to promote information exchange are available routinely to all clinicians who manage patients with anaphylaxis. Brief, psychologically informed training can be an effective way of supporting staff to explore the range of barriers and facilitators related to AAI adherence among patients with anaphylaxis. The *Management Plan* and *AAI Training Checklist,* as developed in this study, could be used to enhance patient AAI training directly (eg, to structure the health encounter with a patient) and indirectly (eg, as a training aide memoire for clinicians before their health encounter with a patient).

This study suggests that clinicians who encounter patients with anaphylaxis at all points of the care pathway should not assume that clinicians other than themselves will train these patients in AAI use. There are some staff for whom training of this nature would be particularly helpful. Staff working in emergency departments may be the first point of contact during, or following, an anaphylaxis emergency, so have an important role to play in the provision of early information about anaphylaxis, including prevention. Specialist allergy staff also have an important role in the provision of practical and psychological information about the impact of anaphylaxis over the longer‐term, particularly for those with adult‐onset. In terms of longer‐term management of anaphylaxis, clinicians based in primary care services have an ongoing role to play in relation to the exploration, and response to, any patient barriers to AAI adherence. For pharmacists, prescription renewal may provide a trigger for effective information exchange about AAI use, including the exploration of intentional and unintentional nonadherence. Given the benefits from whole team training, team workshops would provide new opportunities to skill up staff knowledge and experience in this area, with a “train the trainer” approach encouraging input from experienced colleagues who could share their experiences of implementing new techniques in practice. Reflections amongst colleagues with direct experience of utilising the workshops tools should encourage discussion about the time required for information exchange of this nature, to help colleagues consider how to overcome any anxieties about time poverty.

Although the study produced several promising findings, there were some limitations to this study. The sample was self‐selected. It was possible that the clinicians who volunteered to attend the training did so because they felt positively about the training before attending the workshop. The sample size was relatively small with some attrition over time. However, the attrition rate was not dissimilar to that found in other studies with clinicians that have used online data collection methods that are similar to that used in the quantitative element of this study. The study captured perceived impact on practice rather than measuring any actual changes to behaviour in practice. Future studies would benefit from evaluating actual changes to clinical practice in identified practice areas. In addition, the impact of training on the exchanges between clinicians and patients would be a helpful focus for future research and could inform further developments to the training workshop materials.

## 5 CONCLUSION

Findings from this study suggest that a psychologically informed training workshop for clinicians, with accompanying tools for practice, increases clinician awareness about the psychological consequences of anaphylaxis. Further research is needed to understand more about patient behaviours (intentional and unintentional) which reduce the likelihood of AAI adherence amongst adults, for targeting through training to encourage effective patient and staff information exchange.

## CONFLICT OF INTERESTS

ALK‐Abelló Ltd provided sponsorship for delivery of the training programme and have also sponsored SOH for unrelated projects. Remaining authors declare no conflict of interests. The authors declare that there are no conflict of interests.

## DATA ACCESSIBILITY

The data that support the findings of this study are available from the corresponding author upon reasonable request.

## Supporting information

Supporting information
